# Serum miR-375 Levels Are Closely Related to Disease Progression from HBV Infection to HBV-Related Hepatocellular Carcinoma

**DOI:** 10.1155/2020/5819385

**Published:** 2020-04-20

**Authors:** Weilu Zhang, Ting Fu, Zhenjun Guo, Ye Zhang, Lei Zhang, Haixia Su, Yong Long, Zhaohua Ji, Yongping Yan, Zhongjun Shao

**Affiliations:** ^1^Department of Epidemiology, Ministry of Education Key Lab of Hazard Assessment and Control in Special Operational Environment, School of Public Health, The Air Force Medical University, No. 17, Changle West RD, Xi'an 710032, China; ^2^Shaanxi Provincial People's Hospital, No. 256, Youyi West RD, Xi'an 710068, China; ^3^Center for Infectious Diseases, Tangdu Hospital, Air Force Medical University, 569 Xinsi Rd, Xi'an 710032, China

## Abstract

**Background:**

There is an urgent need to identify ideal serological biomarkers that not only are closely related to disease progression from hepatitis B virus (HBV) infection to hepatocellular carcinoma (HCC) but also have high specificity and sensitivity. We conducted this study to analyze whether miR-375 has a potential value in the early prediction of the progression from HBV-related hepatitis or cirrhosis to HCC.

**Methods:**

A total of 177 participants were enrolled. Receiver operating characteristic (ROC) curve was used to evaluate the predictive capability of selected miR-375 for HBV-HCC. We upregulated the miR-375 expression in HepG2, HepG2.2.15, and HepAD38 cells to determine its effect on cellular proliferation and migration, in vitro using Cell Counting Kit-8 (CCK-8) assays.

**Results:**

Serum miR-375 levels decreased in order from healthy controls to chronic hepatitis B (CHB) without cirrhosis, followed by cirrhosis, and finally, HBV-HCC patients. miR-375 levels were significantly lower in HBeAg-positive and HBV DNA-positive patients than negative (*P* < 0.05) and significantly lower in patients with elevated alpha-fetoprotein (AFP) and carcinoembryonic antigen (CEA) than normal levels (*P* < 0.05). miR-375 might be a biomarker for HBV-HCC, with a high area under the curve (AUC) of 0.838 (95% confidence interval (CI) 0.780 to 0.897; sensitivity: 73.9%; specificity: 93.0%). The AUC (0.768 vs. 0.584) and sensitivity (93.8% vs. 75.0%) for miR-375 were higher than those for AFP. The overexpression of miR-375 noticeably inhibited proliferation and migration in HepG2, HepG2.2.15, and HepAD38, especially in HepG2.2.15 and HepAD38, which are stably infected with HBV.

**Conclusions:**

Serum miR-375 levels are closely related to disease progression from HBV-related hepatitis or cirrhosis to HCC.

## 1. Background

It is estimated that almost 240,000,000 people are chronically infected with hepatitis B virus (HBV). Annually, HBV infection accounts for approximately 1 million deaths [1]. Hepatocellular carcinoma (HCC) is the major form of primary liver tumor, representing the third leading cause of cancer-related death worldwide. Nearly half of HCC cases and deaths are estimated to occur in China due to the high prevalence of HBV infection. The cancer statistics in China show that the mortality due to HCC is 42.21/100,000 [2]. Patients with HCC have a poor prognosis, with a 5-year survival of approximately 5% because of diagnosis at late stages and limited treatment options [3]. Therefore, it is important to predict the progression from HBV-related hepatitis or cirrhosis to HCC.

Recently, many biomarkers have been widely used for HCC diagnosis, such as alpha-fetoprotein (AFP) [4], suppressor with morphogenetic effect on genitalia-1 (SMG-1) [5, 6], Golgi membrane protein 1 (GOLM1) [7], BarH-like Homeobox 2 (Barx2) [8], des-*γ*-carboxy prothrombin (DCP) [9], glypican-3 [10], cytokeratin-19 [11], Golgi protein-73 [12], osteopontin [13], miR-122, and PIVKA-II [14]. Nevertheless, most of these proteins are usually detected after HCC occurrence and lack sensitivity and specificity [15]. Moreover, these markers are not specific for HBV-HCC. Hence, it is still difficult to predict disease progression from healthy liver, chronic hepatitis B infection (CHB) without cirrhosis, and CHB with cirrhosis to HBV-HCC. Therefore, there is an urgent need to identify ideal serological biomarkers that not only are closely related to disease progression from HBV infection to HCC but also have high specificity and sensitivity.

MicroRNAs (miRNAs) are known to play fundamental roles in the regulation of many oncogenes or tumor suppressors and are considered potential diagnostic biomarkers for cancer detection [16, 17]. miR-375 is encoded by the chromosomal region 2q35 in humans. Some studies have reported that miR-375 is downregulated including HCC [18–21]. Research has shown that miR-375 could be used as a serological marker for HCC [22]. When employed as biomarkers, miR-25, miR-375, and let-7f could separate HCC patients from controls. The area under the receiver operating characteristic (ROC) curve (AUC) for miR-375 for HCC prediction was 0.96 (specificity: 96%; sensitivity: 100%) [23]. Mechanistic studies have shown that miR-375 inhibited proliferation, migration, and invasion in Hep3B and Huh7 cells [24]. However, there are very few studies on the relationship between miR-375 and HBV-HCC in patients or in HBV-infected cell lines. Moreover, there is a lack of epidemiological studies on the association of serum miR-375 expression with disease progression from healthy liver, CHB without cirrhosis, and cirrhosis to HBV-HCC.

In this study, we determined serum miR-375 levels in healthy controls and in patients with CHB without cirrhosis, cirrhosis, or HBV-HCC and analyzed the correlation between miR-375 and disease progression, HBV-related serological markers, and clinical features. Then, we upregulated miR-375 expression in HepG2, HepG2.2.15, and HepAD38 cells to determine its effect on cellular proliferation and migration *in vitro*. Additionally, we evaluated the diagnostic value of serum miR-375 as an ideal serological biomarker for the prediction of HBV-related hepatitis or cirrhosis to HCC.

## 2. Materials and Methods

### 2.1. Subjects

From August 2015 to August 2017, healthy individuals and patients with HBV-related diseases who were older than 18 years were recruited from the Department of Hepatobiliary Surgery, Xijing Hospital, and the Center of Infectious Diseases, Tangdu Hospital, Xi'an City, Shaanxi Province, China. All patients were positive for HBsAg, and none of the patients had any other type of liver disease, such as chronic hepatitis C infection, alcoholic liver disease, autoimmune liver disease, or metabolic liver disease. The diagnosis of HCC and cirrhosis was histopathologically confirmed. CHB in the liver was caused by persistent infection with HBV. The diagnostic criteria included the following four points: (1) HBsAg^+^ > 6 months, (2) serum HBV DNA > 20,000 IU/mL (10^5^copies/mL), (3) persistent or intermittent elevation in alanine transaminase (ALT)/aspartate aminotransferase (AST) levels, and (4) liver biopsy showing chronic hepatitis with moderate or severe necroinflammation. All the healthy controls were from the medical examination center, and they were not found to have any disease after physical examination.

A standard questionnaire was used by trained investigators to collect information from face-to-face interviews, pathology reports, and medical records. The questionnaire recorded the following information: basic demographic characteristics of the subjects, family history of HBV infection, cause of disease, tumor pathology, and clinical indicators of cancer. Blood samples for miRNA detection were collected in tubes and processed within 1 h of collection. The blood samples were centrifuged at 12,000 g for 10 min at 4°C to spin down blood cells, and the supernatants were transferred into microcentrifuge tubes, followed by a second centrifugation at 12,000 g for 10 min at 4°C. The supernatants were transferred to RNase-free tubes and stored at −80°C.

### 2.2. Ethics, Consent, and Permissions

The Ethics Committee of the Air Force Medical University approved the study protocol. All participants were fully informed of the details of research study, and the participants or legal guardians of the participants signed written informed consent before inclusion in the study.

### 2.3. Serological Examination of HBV

Blood samples of participants were obtained by forearm venipuncture. The serum samples were laboratory tested after collection. The same lot of enzyme-linked immunosorbent assay (ELISA) reagents from Wantai Production Company (Beijing, China) was used for the initial testing of HBsAg, HBeAg, anti-HBs, anti-HBe, and anti-HBc. All specimens were evaluated for the presence of HBV DNA by using a diagnostic kit (for PCR-based fluorescence probing) according to the instructions of the manufacturer (Roche Molecular Systems). Viral loads higher than a linear range were determined by dilution as recommended by the manufacturer.

### 2.4. Detection of miR-375 via Real-Time Quantitative RT-PCR

#### 2.4.1. RNA Isolation

Total RNA was isolated from serum samples using TRIzol reagent (Invitrogen, USA). Briefly, 250 *μ*L serum samples and all cell lines were used to extract total RNA. Each sample was eluted in 50 *μ*L of RNase-free water. The concentration and purification of RNA were spectrophotometrically determined by measuring its optical density (OD, A260/280>2.0 and A260/230>1.8) using a NanoDrop ND-2000 Spectrophotometer (Thermo Scientific Wilmington, DE, USA).

#### 2.4.2. RNA Reverse Transcription and qRT-PCR

The primers used for Polymerase Chain Reaction (PCR) were as follows: miR-375 forward primer (5′-TTTGTTCGTTCGGCTCGC-3′) and reverse primer and U6 (5′-CGCTTCGGCAGCACATATAC-3′) purchased from TaKaRa (Dalian, China). We used U6 as an endogenous control for both serum and cells. Reverse transcription of miRNAs was performed with the SYBR PrimeScript miRNA RT-PCR Kit (TaKaRa, Dalian, China). The expression of mature miRNAs was determined using miRNA-specific qRT-PCR (TaKaRa, Dalian, China). Fluorescence was automatically detected during amplification, and the melting curve for the product was obtained. The comparative cycle threshold (Ct) method was used for calculation. And the 2^–*Δ*Ct^ method was used to calculate the relative levels of specific molecules [25].

### 2.5. Cell Culture

The following HCC cell lines were used in this study: HepG2, HepG2.2.15, and HepAD38. These cell lines were cultured in Dulbecco's modified Eagle's medium (DMEM, HyClone, USA) supplemented with 10% fetal bovine serum (FBS, Gibco, USA) and 380 mg/L G418 sulfate (Promega, USA) at 37°C in an atmosphere with 5% CO_2_. HepAD38 cells, which are a variant of HepG2 cells, express the HBV genome under the control of a tetracycline- (Tet-) off promoter.

### 2.6. Cell Transfection

miR-375 mimics and negative control (NC) oligonucleotides were designed and synthesized by GenePharma (Shanghai, China). The sequences were as follows: miR-375 mimic, 5′-UUUGUUCGUUCGGCUCGCGUGA-3′ and random miRNA mimics (NC), 5′-UUCUCCGAACGUGUCACGUTT-3′. miR-375 mimics were transfected into cells at a final oligonucleotide concentration of 20 nmol/L. Transfection was performed with Lipofectamine 2000 reagent (Invitrogen, CA, USA) following the manufacturer's protocol. Briefly, cells were trypsinized, counted, and seeded in plates on the day before transfection to ensure suitable cell confluency on the day of transfection.

### 2.7. Proliferation Assay

The Cell Counting Kit-8 (CCK-8) assay was carried out to assess cell viability. HepG2 cells, HepG2.2.15 cells, and HepAD38 were seeded in 96-well culture plates at 2 × 10^5^ cells per well. The next day, the cells were transfected with miR-375 mimics or NC as above. Then, 10 mL of CCK-8 reagent was added to each well before transfection and at 24 h, 48 h, or 72 h after transfection. Cell proliferation rates were determined by the measurement of optical density (OD) at 490 nm via a microplate reader (Bio-Rad).

### 2.8. Statistical Analysis

RT-PCR was performed in triplicate, and average Ct values were calculated. The average expression level of miR-375 in all samples was normalized using U6 as the reference, and the 2^-*Δ*Ct^ method (*Δ*Ct = miR-375 Ct-U6 Ct) was used to express the level of miR-375 in serum samples. The normalized data were analyzed using *t*-test or ANOVA, with asymptotic *P* value computations at *P* < 0.05. Groups of patients with different miR-375 levels in their sera were evaluated by the two-tailed nonparametric Mann–Whitney *U* test. Furthermore, Spearman correlation was used to analyze the correlation between the expression level of miR-375 and patient characteristics. ROC curve analysis was employed to determine the diagnostic utility of miR-375. For cytological experiments, differences among experimental groups were statistically evaluated using *t*-tests. Statistical analyses were performed using SPSS version 19.0 (IBM, USA). All graphs were constructed using GraphPad Prism 5 (GraphPad Software, USA).

## 3. Results

### 3.1. Description and Clinical Features of Patients

A total of 177 participants were enrolled in the study at the Department of Hepatobiliary Surgery, Xijing Hospital and the Center of Infectious Diseases, Tangdu Hospital from August 2015 to August 2017. A total of 63 HBV-HCC patients, 74 patients with CHB, and 40 healthy controls were enrolled in this study. Among the 74 patients with CHB, 54 had cirrhosis, including 20 patients with compensated cirrhosis and 34 patients with decompensated cirrhosis. The average age of all the subjects was 47.24 ± 10.72 years. The average age of the healthy controls and HBV-infected subjects was 45.63 ± 9.43 years and 48.99 ± 10.93 years, respectively, 80% (32/40) of the males in healthy controls and 81% (111/137) of males with those infected with HBV. There was no significant difference in the distribution of age and sex between the above two groups (*P* > 0.05). All patients were positive for HBsAg, and 62 patients were positive for HBV DNA. In addition, 1.5% of the patients were positive for anti-HBs antibodies, 39.4% were positive for HBeAg antibodies, 40.9% were positive for anti-HBe antibodies, and 92.7% were positive for anti-HBc antibodies. There were 74 patients with elevated ALT levels. The tumor markers alpha-fetoprotein (AFP), carcinoembryonic antigen (CEA), and carbohydrate antigen 19-9 (CA19-9) were elevated in 72, 32, and 51 patients, respectively. None of the patients had any other type of liver disease, such as chronic hepatitis C infection, alcoholic liver disease, autoimmune liver disease, or metabolic liver disease.

### 3.2. Expression of miR-375 in Serum

**Figure 1:**
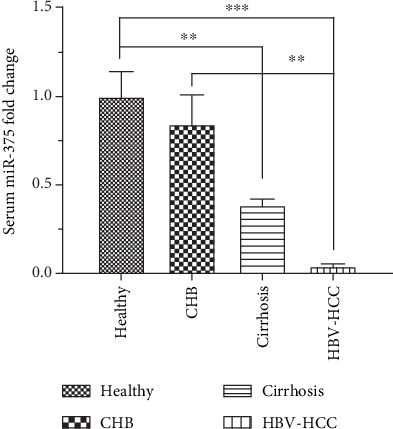
The differential serum levels of miR-375 in the HCC, cirrhosis, CHB, and healthy control groups. The expression of serum miR-375 decreased in order from healthy controls to patients with chronic hepatitis B infection (CHB) without cirrhosis, followed by patients with CHB with cirrhosis (cirrhosis), and finally, patients with HBV-HCC. Serum miR-375 levels were significantly higher in healthy controls than in cirrhosis patients (^∗∗^*P* < 0.01). Serum miR-375 levels were significantly higher in healthy controls than in HBV-HCC patients (^∗∗∗^*P* < 0.001). Serum miR-375 levels were significantly higher in patients with CHB than in patients with HBV-HCC (^∗∗^*P* < 0.01).

To investigate whether serum miR-375 levels are abnormally altered in healthy individuals, in patients with CHB with or without cirrhosis, and in patients with HCC, serum miR-375 levels were measured in 137 patients and 40 healthy controls. The expression of serum miR-375 by qRT-PCR analysis decreased in order from healthy controls to patients with CHB without cirrhosis, followed by patients with cirrhosis, and finally, patients with HBV-HCC. Compared with those in healthy controls, serum miR-375 levels were significantly decreased in cirrhosis and HBV-HCC patients (*P* < 0.05). Among the HBV-infected patients, serum miR-375 levels were significantly higher in the CHB group than those in the HBV-HCC group (*P* < 0.05) (Figure 1).

### 3.3. Relationship between Serum miR-375 Levels and HBV Serological Markers in HBV-Infected Patients

To assess whether serum miR-375 levels were associated with viral replication-related and HBV serological markers, the correlation between serum miR-375 levels and concentrations of HBV DNA (45.26%), HBeAg (39.42%), anti-HBe (40.88%), anti-HBc (92.70%), and anti-HBs (1.46%) antibodies were analyzed in HBV-infected patients. The expression of miR-375 was significantly lower in HBeAg-positive patients than that in HBeAg-negative patients (*P* < 0.05). The expression of miR-375 was significantly lower in anti-HBe-negative patients than that in anti-HBc-positive patients (*P* < 0.05). Additionally, the expression of miR-375 was significantly lower in HBV DNA-positive patients than that in HBV DNA-negative patients (*P* < 0.05) (Table 1).

### 3.4. Relationship between Serum miR-375 Levels and the Clinical Features of HBV-Infected Patients

We next analyzed the correlation between the expression of serum miR-375 and clinical features of patients, such as ALT, AFP, carbohydrate antigen 19-9 (CA19-9), and carcinoembryonic antigen (CEA) levels. The expression of miR-375 was significantly lower in patients with elevated AFP and CEA levels than that in patients with normal AFP and CEA levels (*P* < 0.05, Table 2).

### 3.5. Diagnostic Value of Serum miR-375 for HBV-HCC

**Figure 2:**
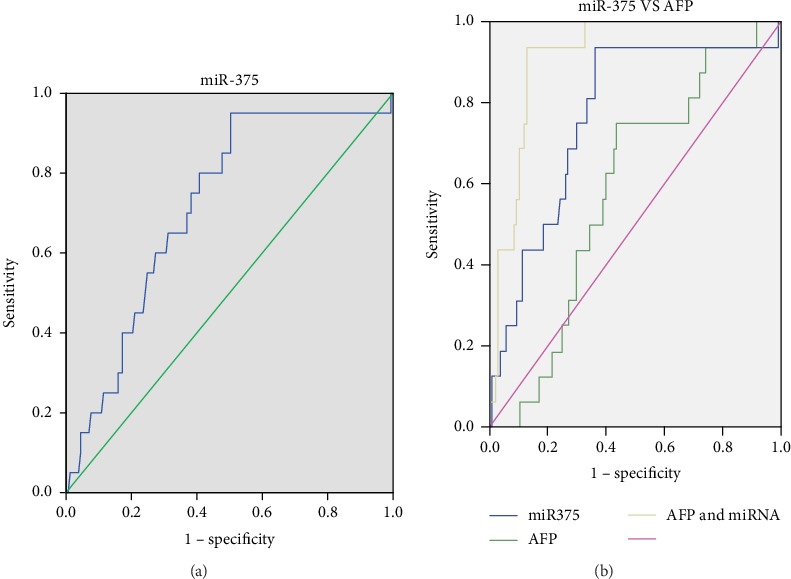
ROC curve analysis of the diagnostic value of serum miR-375 for HCC. (a) AUC estimation for the value of serum miR-375 in identifying HCC patients within the whole cohort (AUC = 0.838, sensitivity = 73.9%, and specificity = 93.0%). (b) AUC estimation of the value of serum miR-375 in distinguishing HCC patients from HBV patients (AUC = 0.768, sensitivity = 93.8%, and specificity = 63.9%). AUC estimation of the value of AFP in distinguishing HCC patients from HBV patients (AUC = 0.584, sensitivity = 75.0%, and specificity = 65.5%).

ROC curve analysis was performed to verify the accuracy of serum miR-375 in diagnosing HBV-HCC. Indeed, serum miR-375 levels could serve as valuable biomarkers for differentiating HCC patients from the whole cohort with an AUC of 0.838 (95% confidence interval (CI): 0.780-0.897, Figure 2) and sensitivity and specificity of 73.9% and 93.0%, respectively. We compared the diagnostic accuracy of miR-375 and AFP using the same serum samples (from 137 HBV-infected patients). miR-375 could differentiate HCC patients from other HBV-infected patients with an AUC of 0.768 (95% CI: 0.644-0.891, Figure 2), sensitivity of 93.8%, and specificity of 63.9%. When we used AFP to separate HBV-HCC patients from HBV-infected patients, the AUC was 0.584 (95% CI: 0.456-0.713), the sensitivity was 75.0%, and the specificity was 65.5%.

### 3.6. miR-375 Was Downregulated in HBV-HCC Cell Lines

**Figure 3:**
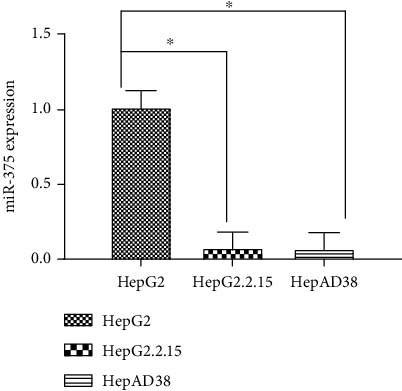
The expression of miR-375 in HepG2, HepG2.2.15, and HepAD38 cell lines. The expression of miR-375 was clearly lower in HepG2.2.15 cells and HepAD38 cells than that in HepG2 cells, respectively. ^∗^*P* < 0.05.

HepG2.2.15, HepAD38, and parental HepG2 cells were selected for in vitro experiments. All cell lines were adjusted to 2 × 10^5^/mL and collected to extract miRNA for use. HepG2.2.15 and HepAD38 cells were positive for HBsAg. However, HepG2 cells were negative for HBsAg, and HBV DNA copy numbers in these cells were below the level of detection. Based on qRT-PCR analysis, miR-375 expression was clearly lower in HepG2.2.15 cells and HepAD38 than that in HepG2 cells (*P* < 0.05, Figure 3), which is consistent with the results of serological analyses.

### 3.7. miR-375 Inhibited the Proliferation of HBV-HCC Cells

We tested the expression level of miR-375 by qRT-PCR in HepG2 cells, HepG2.2.15 cells, and HepAD38 cells at 48 h after transfection to determine the transfection efficiency. The expression of miR-375 in the transfected cells was significantly higher than that in the nontransfected control cells by nearly 15-20-folds, indicating that the expression of miR-375 in the cells was enhanced after transfection.

**Figure 4:**
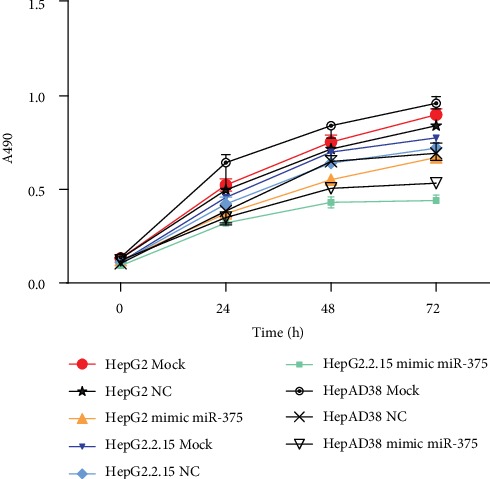
The effect of miR-375 on the proliferation of HCC cells. We performed the CCK-8 assay in HepG2 cells, HepG2.2.15 cells, and HepAD38 cells at 24 h, 48 h, and 72 h after transfecting the cells with miR-375 mimics. HepG2.2.15 and HepAD38 cells were positive for HBsAg. However, HepG2 cells were negative for HBsAg. The proliferation rates of miR-375-transfected cells were lower than those of nontransfected cells (transfected vs. nontransfected HepG2.2.15 cells, transfected vs. nontransfected HepAD38 cells, and transfected vs. nontransfected HepG2 cells). The proliferation rates of HepG2.2.15 cells and HepAD38 cells were lower than those of HepG2 cells (transfected HepG2.2.15 cells vs. transfected HepG2 cells, transfected HepAD38 cells vs. transfected HepG2 cells, and nontransfected HepG2.2.15 cells vs. nontransfected HepG2 cells).

We performed the CCK-8 assay in HepG2 cells, HepG2.2.15 cells, and HepAD38 cells at 24 h, 48 h, and 72 h after transfecting the cells with miR-375 mimics. The proliferation rates of miR-375-transfected cells were lower than those of nontransfected cells (transfected vs. nontransfected HepG2.2.15 cells, transfected vs. nontransfected HepAD38 cells, and transfected vs. nontransfected HepG2 cells). The proliferation rates of HepG2.2.15 cells and HepAD38 cells were lower than those of HepG2 cells (transfected HepG2.2.15 cells and HepAD38 vs. transfected HepG2cells, nontransfected HepG2.2.15 and HepAD38 cells vs. nontransfected HepG2 cells). The proliferation rates of transfected HepG2.2.15 cells and HepAD38 were the lowest among all groups. Therefore, the overexpression of miR-375 significantly inhibited cellular proliferation (Figure 4).

## 4. Discussion

The purpose of the present study was to explore novel serum biomarkers for the prediction of HBV-HCC progression. Because of the lack of reliable serum biomarkers with high sensitivity and specificity, we conducted the present study to identify whether miR-375 has a potential diagnostic value in predicting HBV-related hepatitis or cirrhosis to HCC.

First, we analyzed serum miR-375 levels in healthy controls and in patients with CHB, cirrhosis, or HBV-HCC. Compared with those in healthy controls, serum miR-375 levels were significantly decreased in cirrhosis and HBV-HCC patients (*P* < 0.05). These results were consistent with our previous study, which compared miR-375 expression in tumor and adjacent normal tissues from patients with HBV-HCC [26]. In that study, miR-375 levels were significantly lower in tumor tissues than in adjacent tissues from HBV-HCC patients. In other words, miR-375 expression is downregulated in both tissues and sera of HBV-HCC patients. Previous studies have also demonstrated that miR-375 is significantly downregulated in multiple types of cancer and it plays the role of a tumor suppressor [3, 18]. Moreover, our data showed that the expression of serum miR-375 decreased in order from healthy controls to patients with CHB without cirrhosis, followed by patients with cirrhosis, and finally, patients with HBV-HCC. Serum miR-375 expression was the lowest in the HBV-HCC group. Therefore, the expression of serum miR-375 decreases with disease progression. With the identification of the potential target genes of these HCC-associated miRNAs, it may shed light into our understanding of tumorigenesis and development of HCC. For example, serum miR-375 contributed to separation of both HBV-infection cases and HBV-positive HCC cases from the controls.

Next, we analyzed the relationship between serum miR-375 levels and HBV serological markers in HBV-infected patients. Serum miR-375 expression was significantly lower in HBeAg-positive and HBV DNA-positive patients than that in HBeAg-negative and HBV DNA-negative patients. HBV DNA is a quantitative virologic marker reflecting the degree of replication of HBV. A number of studies have found that the amount of HBV DNA is related to the extent of liver injury and severity of liver fibrosis and that it can be used as an independent factor to predict response to antiviral treatment [26–28]. HBeAg is a serologic marker associated with a high degree of viral replication and infectivity. In general, HBeAg positivity is correlated with a high HBV DNA load [29]. In this study, the expression of serum miR-375 was relatively lower in HBV DNA-positive and HBeAg-positive patients. The results of our previous study showed that the abnormal expression of miR-375 in HBV-HCC patient tissues was closely related to the replication status of HBV. In addition, the expression level of miR-375 in tissues was negatively correlated with the HBV DNA load. The higher was the titer of HBV DNA, the lower was the expression level of miR-375 in tissues [26]. These results were consistent with the results of the current study.

Next, we analyzed the relationship between serum miR-375 levels and commonly used serum tumor markers. AFP is one of the most widely used tumor markers for the diagnosis of HCC, and it has a sensitivity of 60% at a cutoff value of 20 ng/mL [4, 27]. A systematic review evaluating AFP (at a threshold level of 20 ng/mL) in cirrhotic patients showed sensitivities and specificities of 41% to 65% and 80% to 94%, respectively, for HCC at any stage [30]. However, at this threshold, early stage HCC was detected in only one-third of patients with the disease [31]. The problem with AFP as a reliable HCC biomarker is that HCC is positive for the protein in only 60%-80% of cases, and false positives make it difficult to distinguish early stage HCC from other disorders, such as acute hepatitis and cirrhosis, as well as embryonic tumors and certain gastrointestinal tumors. Thus, in order to significantly improve the diagnostic accuracy for HCC, additional biomarkers are needed to complement AFP, especially due to the fact that many patients with benign liver diseases, such as chronic hepatitis, liver cirrhosis, and gastrointestinal cancer, also have elevated serum AFP. To compare the diagnostic accuracy of miR-375 and AFP, we analyzed the AUC of the two markers using the same serum samples. The AUC (0.768 vs. 0.584) and sensitivity (93.8% vs. 75.0%) of serum miR-375 were higher than those for AFP. Therefore, serum miR-375 is better than AFP for diagnosing HBV-HCC. Nevertheless, these results need to be validated in larger cohorts in the future.

In recent years, some studies have shown that abnormal expression of miR-375 can affect the proliferation, migration, and invasion of HCC cell lines, including Hep3B and Huh7 [24, 32]. However, these studies determined the expression of miR-375 and its downstream regulatory genes in pure hepatoma cell lines but not HBV-HCC cell lines. Therefore, the effect of the abnormal expression of miR-375 on the biological function of HBV-HCC cell lines remained unknown. The cell lines HepG2.2.15 and HepAD38 that were used in this study have been derived by the stable expression of HBV in HepG2 cells. We routinely cultured and confirmed the identity of HepG2, HepG2.2.15, and HepAD38 cells. HepG2.2.15 and HepAD38 cells were positive for HBsAg, and HBV DNA titers in these cells indicated moderate viral replication, whereas HepG2 cells were negative for HBsAg and HBV DNA. Therefore, HepG2.2.15 and HepAD38 displayed characteristics of HBV infection, and there was no cross contamination between the three cell lines.

In vitro, we determined the expression level of miR-375 in HepG2.2.15, HepAD38 cells, and parental HepG2 cells using qRT-PCR. Compared with that in HepG2 cells, which are not infected with HBV, the expression level of miR-375 was clearly reduced in HepG2.2.15 cells and HepAD38, which are infected with HBV. In other words, the expression level of miR-375 in liver cancer cells is affected by HBV infection. Some studies have also shown that other miRNAs are differentially expressed between cell lines with stable HBV infection and their parental counterparts [26]. miRNAs play a critical role in virus-host interactions. Cellular miRNAs in the host modulate the expression of various viral genes, thus playing a pivotal role in the host-pathogen interaction network. In addition, viruses also encode miRNAs for protection against cellular antiviral responses or may even exploit host miRNA pathways to their own advantage. The persistence of the virus in host cells in turn affects the expression of miRNAs in the host.

We upregulated miR-375 expression in HepG2, HepG2.2.15, and HepAD38 cells and analyzed the effects of miR-375 overexpression on proliferation and invasion in vitro using the CCK-8 and transwell assays. The overexpression of miR-375 significantly inhibited cellular proliferation. In HepG2, HepG2.2.15, and HepAD38 cells, the proliferation rate of miR-375-transfected cells was lower than that of nontransfected cells. Maximum inhibition was observed at 48 h posttransfection. Additionally, the proliferation rates of HepG2.2.15 cells and HepAD38 with or without miR-375 overexpression were significantly lower than the respective proliferation rates of HepG2 cells with or without miR-375 overexpression. Between 48 and 72 h posttransfection with miR-375, the size of the HepG2.2.15 cell population remained almost constant. Therefore, miR-375 could inhibit the proliferation of liver cancer cells, and the inhibition was more pronounced in the presence of HBV infection.

In other words, miR-375 plays a similar tumor suppressor gene. Based on past experiments, JAK-2 acts as a downstream target of miR-375 and is regulated by miR-375, which affects the normal expression of genes and proteins in the signal transduction pathway and affects the proliferation and invasion of tumor cells. Similarly, YAP1 is also an important target molecule of miR-375, which is an important molecule in the hippo signaling pathway. It can promote proliferation and growth and plays an important role in DNA repair. When the hippo pathway abnormal YAP1 is activated, YAP1 is a famous oncoprotein, and the incidence of cancer is closely related to the development of the tumor can promote. miR-375 can reverse the inhibition of YAP1 expression, so that YAP1 to promote the tumor was cut off to this channel to achieve the purpose of inhibiting tumorigenesis [33]. Because miR-375 likely serve a role in HCC development following chronic HBV infection, future study may focus on their target genes and the mechanisms by which they execute their functions during HBV infection and HBV-positive HCC development.

The present study has two limitations. First, during the transition from healthy liver, HBC, and liver cirrhosis to HBV-HCC, there is an asymptomatic phase of HBV infection. However, our cohort did not include such HBV carriers. In the future, we will further expand the sample size and evaluate patients at various stages of HBV infection, including chronic asymptomatic HBV carriers. Thus, we will perform in-depth analysis to further confirm the diagnostic value of serum miR-375 for HBV-HCC. Second, this study only examined miR-375, and other potentially relevant markers, including some classic HCC risk factors, could not be evaluated. In the future, we will assess these markers together with miR-375 and construct a predictive model for HBV-HCC.

## 5. Conclusion

Serum miR-375 expression decreased in order from healthy controls to patients with CHB without cirrhosis, followed by patients with CHB with cirrhosis, and finally, patients with HBV-HCC. Serum miR-375 expression was correlated with HBeAg, HBV DNA, and AFP and CEA levels. ROC curve analyses revealed that serum miR-375 may be a promising biomarker for HBV-HCC detection, with a relatively high AUC, sensitivity, and specificity. The AUC and sensitivity of serum miR-375 were higher than those for AFP. In addition, compared with that in HepG2 cells, miR-375 expression was markedly reduced in HepG2.2.15 and HepAD38 cells. The overexpression of miR-375 clearly inhibited proliferation and migration *in vitro*, especially in HepG2.2.15 cells and HepAD38, which are stably infected with HBV. Therefore, serum miR-375 might serve as a potential biomarker to predict the progression of HBV-related hepatitis or cirrhosis to HCC.

## Figures and Tables

**Table 1:** Relationship between serum miR-375 levels and HBV serological markers in the HBV-infected patients (*n*(%)).

HBV serological markers	Number of patients (%)	miR-375 levels	*t* value	*P*
Anti-HBs			0.662	0.509
Positive	2 (1.46%)	0.019 ± 0.016		
Negative	135 (98.54%)	0.125 ± 0.021		
HBeAg			2.037	0.044^∗^
Positive	54 (39.42%)	0.081 ± 0.020		
Negative	83 (60.58%)	0.186 ± 0.047		
Anti-HBe			2.174	0.032^∗^
Positive	56 (40.88%)	0.078 ± 0.020		
Negative	81 (59.12%)	0.164 ± 0.034		
Anti-HBc			0.961	0.339
Positive	127 (92.70%)	0.129 ± 0.022		
Negative	10 (7.30%)	0.058 ± 0.038		
HBV DNA			2.022	0.046^∗^
Positive	62 (45.26%)	0.083 ± 0.023		
Negative	75 (54.74%)	0.196 ± 0.050		

Serum HBV DNA ≥ 10^2^ copies/mL was defined as HBV DNA-positive. Serum HBV DNA < 10^2^ copies/mL was defined HBV DNA-negative. ^∗^*P* < 0.05.

**Table 2:** Relationship between serum miR-375 levels and clinical features (*n*(%)).

Clinical features	Number of patients (%)	miR-375 levels	*t* value	*P*
ALT			0.363	0.717
Elevated	74 (54.0%)	0.103 ± 0.027		
Normal	63 (46.0%)	0.116 ± 0.026		
AFP			3.406	0.001^∗∗∗^
Elevated	72 (52.6%)	0.048 ± 0.015		
Normal	65 (47.4%)	0.252 ± 0.058		
CA19-9			1.100	0.273
Elevated	51 (37.2%)	0.102 ± 0.029		
Normal	86 (62.8%)	0.170 ± 0.044		
CEA			2.221	0.028^∗^
Elevated	32 (23.4%)	0.063 ± 0.030		
Normal	105 (76.6%)	0.170 ± 0.038		

Logistic regression analyses in subgroups of patients: categorized by serum levels of ALT (<40 U/L and ≥40 U/L), AFP (<20 ng/L and ≥20 ng/L), CA19-9 (<37 U/mL and ≥37 U/mL), and CEA (<5 *μ*g/mL and ≥5 *μ*g/mL) at baseline. ^∗^*P* < 0.05, ^∗∗∗^*P* < 0.001.

## Data Availability

All data used to support the findings of this study are included within the article. The raw data in this study are restricted by the Independent Ethics Committee, The Air Force Medical University, China, in order to protect patient privacy. Data are available from the corresponding author: Zhongjun Shao (e-mail: zhongjunshao@126.com) for researchers who meet the criteria for access to confidential data.
